# Protective effects of curcumin on diabetic nephropathy via attenuation of kidney injury molecule 1 (KIM-1) and neutrophil gelatinase-associated lipocalin (NGAL) expression and alleviation of oxidative stress in rats with type 1 diabetes

**DOI:** 10.22038/ijbms.2019.31922.7674

**Published:** 2019-04

**Authors:** Hassan Ghasemi, Behzad Einollahi, Nejat Kheiripour, Seyed-Reza Hosseini-Zijoud, Masoumeh Farhadian Nezhad

**Affiliations:** 1 Department of Clinical Biochemistry, Abadan School of Medical Sciences, Abadan, Iran; 2 Nephrology and Urology Research Center, Baqiyatallah University of Medical Sciences, Tehran, Iran; 3 Research Center for Biochemistry and Nutrition in Metabolic Diseases, Kashan University of Medical Sciences, Kashan, Iran; 4 Faculty of Medicine, Shahid Beheshti University of Medical Sciences, Tehran, Iran; 5 Student Research Committee, Abadan School of Medical Sciences, Abadan, Iran

**Keywords:** Curcumin, Kidney injury molecule 1, Neutrophil gelatinase-associated lipocalin protein, Oxidative stress, Type 1 diabetes mellitus

## Abstract

**Objective(s):**

One of the serious complications of Type1 diabetes (T1D) is diabetic nephropathy, which is accompanied with overexpression of kidney injury molecule 1 (KIM-1) and neutrophil gelatinase-associated lipocalin (NGAL) and enhanced oxidative stress. The present study was conducted to examine the protective effect of curcumin on the expression of KIM-1, NGAL genes and oxidative damage in the kidney of T1D rats.

**Materials and Methods:**

Thirty-six adult male rats were divided into 6 groups (n=6). The control and T1D groups received treatment with curcumin or without it (80 and 130 mg/kg, respectively). After 60 days of treatment, using spectrophotometric methods, biochemical factors and oxidative stress markers were measured. Gene expression of KIM-1 and NGAL was evaluated using quantitative PCR. Also, plasma and urine levels of these two proteins were assayed by the ELISA kit.

**Results:**

Diabetes caused a significant increase in the levels of creatinine, FBS, uric acid, urea, and creatinine in the serum, which were attenuated after the administration of curcumin. There was a significant reduction in the values of creatinine, uric acid, and urea in urine in the diabetic group whereas in the rats treated with curcumin, these values were normalized to the normal level (especially in 130 mg/kg). Curcumin administration had a significant role in modulation of serum lipid profile, and it was shown to decrease the kidney and urinary expression levels of KIM-1 and NGAL genes and improve oxidative toxic stress in the kidney tissues.

**Conclusion:**

Curcumin can play a protective role in reducing the unpleasant consequences of diabetic nephropathy.

## Introduction

Diabetes mellitus is a chronic metabolic disorder marked by hyperglycemia and defect in the metabolism of carbohydrates, lipids, and proteins (1). The number of people around the world who are affected by this disorder is approximately 415 million according to a report released by the International Diabetes Federation (2). Of all cases of diabetes, around 5–10% are of Type 1 diabetes (T1D), an autoimmune disease in which the pancreatic beta cells are destroyed and there is an insufficient level of insulin (3, 4).

One of the serious microvascular complications of T1D is diabetic kidney disease that can result in end-stage renal disease (5, 6). According to previous studies, a number of factors are involved in the pathogenesis of diabetic kidney disorder, namely hexosamine, protein kinase C (PKC), formation of advanced glycation end products (AGEs) and polyol pathways (7). A common characteristic of all these pathways is an imbalance between the production and degradation of free radicals called oxidative stress (8). Diabetic hyperglycemia has an important role in oxidative stress through increased levels of reactive oxygen species (ROS) (6). More specifically, diabetic hyperglycemia activates the protein kinase C pathway (PKC) in glomerulus through diacylglycerol (DAG), consequently causing thickening of the glomerular membrane and renal dysfunction (9). Therefore, diabetic hyperglycemia-induced oxidative stress seems to play an important role in diabetic kidney disorder.

Kidney injury molecule 1 (KIM-1) is a transmembrane glycoprotein that is up-regulated after acute ischemic kidney injury in the proximal tubule of renal tissue (10, 11). It is a specific and sensitive biomarker in the diagnosis of kidney injury based on the reports of the Food and Drug Administration (FDA) (12). 

Neutrophil gelatinase-associated lipocalin protein (NGAL) as a member of lipocalin superfamily, is a 25-kDa glycoprotein that was first detected in human activated neutrophils (13). In normal conditions, NGAL is expressed and secreted by the kidneys, trachea, lungs, stomach, and colon at a very low level, and is completely reabsorbed after glomerular filtration (14). Under kidney tubular dysfunction caused by kidney injury, NGAL is overexpressed and secreted in urine (15). Previous studies have indicated that NGAL may be an appropriate biomarker for assessment of kidney injury in diabetic patients (16, 17).

Studies on the role of antioxidant herbal compounds in the treatment of diabetes complications are among the issues of interest to researchers. Curcumin is a phenolic compound derived from the rhizome of the herb *Curcuma longa* and has many therapeutic benefits in different diseases ranging from cancer to cystic fibrosis (18). Previous studies suggest that curcumin has a potent antioxidant activity compared to vitamins C and E (19). Despite the fact that previous studies have already demonstrated the anti-oxidant role of curcumin in diabetes, little is known about how it can affect the kidney injury caused by T1D. Therefore, the aim of the present study was to investigate the antioxidant effects of curcumin on renal expression and urinary levels of KIM-1 and NGAL and also renal oxidative stress in rats having T1D. 

## Materials and Methods

***Animals and experimental designs***

The animals used in this study were 36 adult male Wistar rats (200–240 g). They were kept under standard conditions (12-hr dark/light cycle at 22 ± 2 °C) and then randomly ‎divided into 6 groups (n=6), including; Group C: normal control receiving normal saline, Groups C+Cur80 and C+Cur130: control rats receiving curcumin (Sigma Aldrich) (80 and 130 mg/kg daily, respectively), Group D: diabetic control, Groups D+Cur80 and D+Cur130: diabetic group receiving curcumin (80 and 130 mg/kg daily, respectively). Treatment was carried out by gavage for 60 days. In order to induce Type 1 diabetes, streptozotocin (STZ, Sigma) (60 mg/kg; intraperitoneal (IP) injection) dissolved in citrate buffer (0.1 M, pH: 4.5) was used. To confirm type 1 diabetes, after 72 hr, fasting blood sugar was measured using glucometer (Accuchek; Roche, Germany). Animals were categorized as diabetic if their fasting blood sugar exceeded 250 mg/dl. All procedures of this study were based on the guidelines of the Medical Ethics Committee of Baqiyatallah University of Medical Sciences**.**

***Lipid profile***

The lipid profile including total cholesterol (TC), triglycerides (TG), and high-density lipoprotein cholesterol (HDL-C) was measured using a kit (Pars Azmun, Iran). The levels of low-density lipoprotein cholesterol (LDL-C) were measured by the Friedewald formula (20).

***Urea, uric acid, and creatinine assay***

Spectrophotometric methods using related kits (Pars Azmun, Iran) were used to measure levels of uric acid, creatinine, and urea. 

***Preparation of kidney tissue***

 Kidney tissue samples separated from all animals were rinsed with saline solution and completely powdered with liquid nitrogen and stored at -80 °C. Afterward, the prepared homogenate was re-suspended and incubated with the lysis buffer (HEPES 10 mM; 10 mM KCl, 1.5 mM MgCl2, 1 mM EDTA, 0.1% Triton X100, 0.5 mM Dithiothreitol, protease inhibitor cocktail, pH 7.9) for 20 min. Tissue homogenate was centrifuged (10 min at 2000 g, 4 ^°^C) and then the supernatant was isolated for more analysis (21). 

***Lipid peroxidation measurement***

Lipid peroxidation was measured using the Yagi method (22). This method is based on the reaction of thiobarbituric acid (TBA) with malondialdehyde (MDA) –a lipid peroxidation end product– that produces a red adduct with maximum absorbance at 532 nm. Tetraethoxypropane standard solutions were used as a standard curve to determine the concentrations of TBA+MDA adducts in samples (23).

***Total antioxidant capacity measurement***

The ferric reducing ability of plasma (FRAP) method was used to evaluate the total antioxidant capacity (TAC). This method is an antioxidant capacity assay that measures the ability of biological antioxidants in reducing Fe^3+^ to Fe^2+^ in the presence of TPTZ (2,4,6 tripyridyl-s-triazine). A blue color complex results upon the reaction of Fe^2+^ with TPTZ, the maximum absorbance of which is at 593 nm (24). 

**Figure 1:**
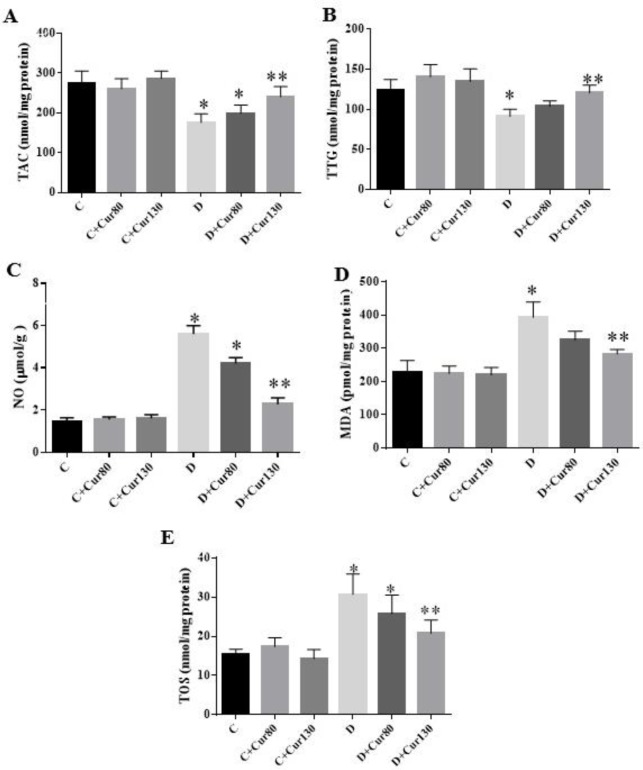
The effect of curcumin on kidney levels of (A) total antioxidant capacity (TAC), (B) total thiol group (TTG), (C) nitric oxide (NO), (D) malondialdehyde (MDA), and (E) total oxidant status (TOS). Data are presented as mean±SD. * Significantly different compared with control groups (*P*<0.05). ** Significantly different compared with diabetic control groups (*P*<0.05)

**Figure 2:**
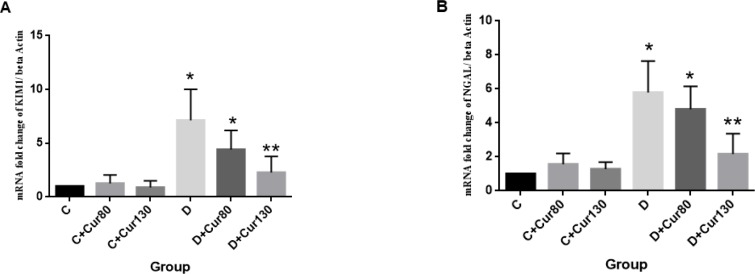
The effect of curcumin on the KIM-1 (A) and NGAL (B) mRNA folding change. The data are presented as mean±SD. C, healthy control; Cur80, curcumin 80 mg/kg; Cur130, curcumin 130 mg/kg; D, diabetic control. * Significantly different compared with control groups (*P*-value <0.05). ** Significantly different compared with control groups (*P*-value <0.05)

**Figure 3:**
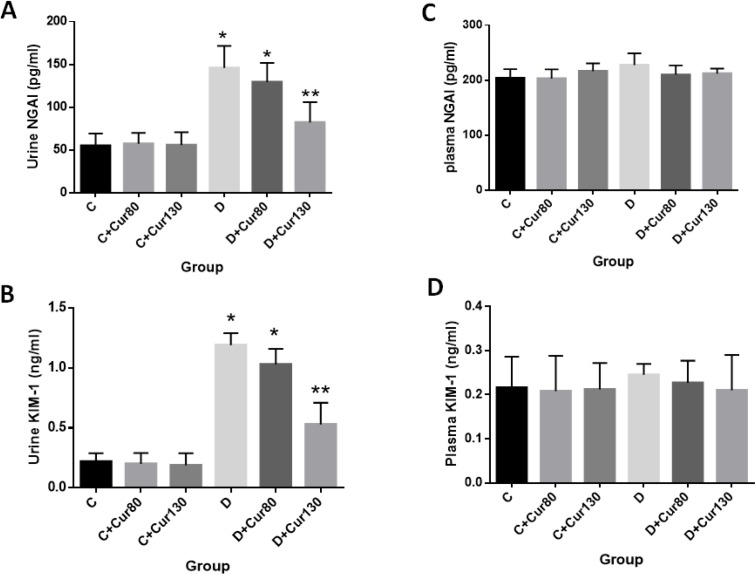
The effect of curcumin on (A) urine NGAL, (B) urine KIM-1, (C) plasma NGAL and (D) plasma KIM-1. Data are presented as mean±SD. C, healthy control; Cur80, curcumin 80mg/kg; Cur130, curcumin 130 mg/kg; D, diabetic control. * Significantly different compared with control groups (*P*-value <0.05). ** Significantly different compared with control groups (*P*-value <0.05)

**Table 1:** The effect of curcumin on the body weight and biochemical parameters of the studied groups

Parameter/ Group	C (n=6)	C + Cur80 (n=6)	C + Cur130 (n=6)	D (n=6)	D + Cur80 (n=6)	D + Cur130 (n=6)
Initial body weight (gr)	228.50±8.8	225.1±7.4	230.3±10.35	227.6±8.2	229.6±6.9	225.8±6.7
Final body weight (gr)	301.50±17.8	292.00±20.5	295.00±18.4	186.50±16.8^a^** #**	219.6±20.3 b** †**	223.8±22.5 b** †**
FBS (mg/dl)	80.8±8.2	77.50±12.2	75.60±9.4	301.3±26.5 a** #**	207.4±34.8 b** #**	185.7±32.4 b** #**
Urea (mg/dl)	20.8±2.5	22.4±2.8	21.3±1.4	51.5±9.5^a^** #**	45.6±6.3 a**†**	28.5±7.1^b^** †**
Creatinine (mg/dl)	0.51±0.06	0.53±0.04	0.48±0.08	0.87±0.07^a^** †**	0.69±0.06 a†	0.52±0.07 b**†**
Uric acid (mg/dl)	2.4±0.30	2.53±0.45	2.48±0.32	6.34±0.7 a** #**	4.8±0.28	3.1±0.2^b^**†**
Total cholesterol (mg/dl)	92.3±6.4	95.3±4.8	90.2±7.3	168.7±10.4 a #	150.2±8.8 a**#**	110.5±11.5 b**†**
Triglyceride (mg/dl)	85.3±4.5	90.3±6.2	82.7±5.4	150.3±9.3 a** #**	135.4±6.7^a^**#**	110.3±6.8 b**†**
HDL-C (mg/dl)	46.7±4.2	45.3±5.1	43.8±3.8	28.3±4.7 a**†**	37.5±6.2	40.1±6.4^ b^**†**
LDL-C (mg/dl)	28.54±5.5	31.94±4.3	29.86±4.4	110.34±6.4 a #	85.62±7.3 a**#**	48.34±5.3 b**†**

Data are presented as mean±SD. HDL-C: High-Density Lipoprotein Cholesterol, LDL-C: Low-Density Lipoprotein Cholesterol, C, healthy control; Cur80, curcumin 80mg/kg; Cur130, curcumin 130 mg/kg; D, diabetic control;

a Significantly different compared with control groups.

b Significantly different compared with diabetic control groups. *P*-value^ #^<0.001, *P*-value^ †^ < 0.01

**Table 2:** The effect of curcumin on urine parameters of the studied groups

Parameter/ Group	C (n=6)	C + Cur80 (n=6)	C + Cur130 (n=6)	D (n=6)	D + Cur80 (n=6)	D + Cur130 (n=6)
Urine volume (ml/day)	14.8±3.2	15.3±2.3	14.4±1.7	64.50±8.3^a^#	48.8±4.2 a #	22.6±2.9 b†
Uric acid (mg/dl)	3.6±0.16	3.54±0.14	3.48±0.12	1.56±0.1a††	1.3±0.14^a^††	2.5±0.22 b††
Creatinine (mg/dl)	25.2±3.3	27.1±2.5	28.36±3.08	9.4±1.4 a†	12.5±2.8 a††	18.16±2.07 b††
Urea(mg/dl)	42.3±3.8	40.5±3.7	38.7±4.1	10.4±2.1 a†	14.9±3.1^a^††	28.7±2.8 b††

Data are presented as mean±SD. C, healthy control; Cur80, curcumin 80mg/kg; Cur130, curcumin 130 mg/kg; D, diabetic control.

a Significantly different compared with control groups.

b Significantly different compared with diabetic control groups. *P*-value

†† <0.05,

† P < 0.01,

# *P*-value <0.001

***Total oxidant status measurement***

Total oxidant status (TOS) was measured using the spectrophotometric method and based on ferrous oxidation by xylenol orange. Under acidic conditions, biological oxidant molecules oxidize ferrous ions to ferric; the ferric ions then react with xylenol orange and produce a colored complex (25).

***Total thiol groups measurement***

Total thiol groups (TTG) are sensitive to oxidative damage and decrease under oxidative stress conditions. To evaluate TTG, DTNB (5,5’-Dithiobis-(2-nitrobenzoic acid)) was used as a reagent. DTNB reacts with the thiol group and produces a yellow complex with maximum absorbance at 412 nm (26). 

***Nitric oxide assay***

Nitric oxide levels were measured using an ELISA kit (ZellBio GmbH, Ulm-Germany), according to the manufacturer’s instructions.

***Quantitative real-time PCR of KIM-1 and NGAL***

Total RNA was extracted from renal tissue of the study groups by RNX- Plus reagent (Cinnagen, Tehran, Iran). Complementary DNA (cDNA) was synthesized by the Prime Script RT reagent kit (TaKaRa Biotechnology, Japan) according to the manufacturer’s instructions. Afterward, Quantitative Real-Time PCR reaction was carried out with SYBR Premix Ex TaqTM II (TaKaRa Biotechnology, Japan) by a Roche Light Cycler 96 System (Roche Life Science Deutschland GmbH, Sandhofer, Germany). Amplification protocol was followed after initial denaturation at 95 ^°^С for 30 sec by 30 cycles (95 ^°^С for 20 sec, 58 ^°^С for 30 sec, and 72 ^°^С for 30 sec). Primer sequences used in this reaction were as follows; NGAL, forward: 5’- GATGAACTGAAGGAGCGATTC- 3’, reverse: 5’-TCGGTGGGAACAGAGAAAAC- 3’, KIM-1, forward: 5’-ACTCCTGCAGACTGGAATGG - 3’, reverse: 5’-ACTCCTGCAGACTGGAATGG - 3’, β-actin, forward: 5’- TCA TTG ACC TCA ACT ACA- 3’, and reverse: 5’- CAAAGTTGTCATGGA TGACC- 3’. The relative copy number of mRNA of target genes was calculated by the 2^-ΔΔCt^ formula (27).

***NGAL and KIM-1 measurement in urine and plasma***

The NGAL and KIM-1 levels in urine and plasma were measured using the ELISA kit (Cusabio Biotech, Wuhan, China), based on the manufacturer’s instructions. 

***Statistical analysis***

The analysis was carried out using SPSS software version 16.0 (SPSS Inc., Chicago-USA) and Graph Pad Prism version 6.0 (Graph Pad Software, San Diego-USA). Data were presented as mean±SD. The statistical differences of the means of variables were evaluated using one-way analysis of variance (ANOVA) followed by Tukey’s test. Data were considered statistically significant when *P*-value < 0.05.

## Results

***General and biochemical measurement***

The weight and biochemical parameters of the studied groups are shown in Table 1. The diabetic groups had a significantly lower final body weight compared with the control groups (*P*-value <0.05). However, treatment with curcumin 80 mg/kg and 130 mg/kg in the diabetic group improved body weight significantly (*P*-value <0.05). There was a significant increase in levels of fasting blood sugar (FBS), urea, uric acid, and creatinine in the diabetic control group compared with the control groups (*P*-value<0.05). In the diabetic group, treatment with curcumin 80 mg/kg and 130 mg/kg caused a significant decrease in the FBS level compared with the diabetic control (*P*-value<0.05). On the other hand, curcumin only in 130 mg/kg dose caused a significant reduction in the serum urea, uric acid, and creatinine levels in the diabetic group (*P*-value <0.05).

The levels of TC, TG, and LDL-C in the diabetic and diabetic control groups treated with curcumin 80 mg/kg were significantly higher than those in the control groups (*P*-value<0.001). Also, the concentration of HDL-C in the diabetic control group significantly decreased in comparison with the controls (*P*-value <0.01). However, in the diabetic group, treatment with curcumin 130 mg/kg significantly decreased the TC, TG, and LDL-C levels (*P*-value<0.01) whereas HDL-C significantly increased (*P*-value <0.01). 

***Effect of curcumin on the oxidative stress markers***

The values of oxidative stress biomarker of renal tissue are summarized in Figure 1. The levels of TAC (Figure 1A) in the diabetic control group and the diabetic group treated with curcumin 80 mg/kg significantly decreased compared to the normal controls (*P*-value = 0.03). Meanwhile, treatment with curcumin 130 mg/kg caused a significant increase in the TAC levels of the diabetic group compared to the diabetic control group (*P*-value = 0.038). 

In diabetes induced by STZ the values of TTG significantly decreased (Figure 1B) compared with the normal control group (*P*-value= 0.032). However, diabetic group treated with curcumin 130 mg/kg, had significantly improved TTG levels (*P*-value= 0.042).

The NO level measurement as an oxidative stress marker showed that its level in the diabetic control group (*P*-value <0.001) and the diabetic group treated with curcumin 80 mg/kg (*P*-value =0.01) increased significantly compared to the normal control (Figure 1C).

MDA assay (Figure 1D) as another oxidative stress indicator showed a significant increase (*P*-value =0.02) in kidney tissue of the diabetic control group compared with the normal control. However, the diabetic group treated with curcumin 130 mg/kg had a significant decrease in MDA concentration compared to the diabetic control group (*P*-value= 0.04). 

Renal TOS in the diabetic control group (*P*-value= 0.01) and the diabetic group treated with curcumin 80 mg/kg (*P*-value =0.03) was significantly higher than that of the normal control group (Figure 1E). TOS concentration in the diabetic group treated with curcumin 130 mg/kg significantly decreased compared to the diabetic control group (*P*-value= 0.035).

***Effect of curcumin on urine parameters***

The results of urine parameters are presented in Table 2. The urine volume in diabetic control and diabetic treated with curcumin 80 mg/kg was significantly higher than that in the control group (*P*-value<0.001). However, in the diabetic group treated with curcumin 130 mg/kg, urine volume significantly decreased compared to the diabetic control group (*P*-value<0.01). The uric acid, urea, and creatinine levels of the diabetic group and those of the diabetic group treated with curcumin 80 mg/kg were significantly lower than those of the control groups (*P*-value<0.05). However, treatment with curcumin 130 mg/kg significantly improved the exertion of these factors (*P*-value<0.05). 

***Effect of curcumin on KIM-1 and NGAL gene expression***

The results of renal NGAL and KIM-1 gene expression are shown in Figure 3 (A and B, respectively). The NGAL gene expression significantly increased in the diabetic control group (*P*-value =0.003) and in the diabetic group treated with curcumin 80 mg/kg (*P*-value <0.03) compared with the controls. Treatment of diabetic group with curcumin 130 mg/kg caused a significant decrease in NGAL gene expression compared to the diabetic control (*P*-value= 0.006). 

The KIM-1 mRNA levels in the diabetic control group (*P*-value <0.001) and the diabetic group that received curcumin 80 mg/kg (*P*-value =0.01) were significantly higher than those in the control groups. On the other hand, treatment with curcumin 130 mg/kg caused a significant decrease in KIM-1 mRNA levels of the diabetic group compared with the diabetic control group (*P*-value<0.001).

***Effect of curcumin on KIM-1 and NGAL levels***

The effect of curcumin on NGAL and KIM-1 levels in plasma and urine was also determined in this study via the ELISA kit. The urine concentrations of NGAL (Figure 3A) and KIM-1 (Figure 3B) in the diabetic group and the diabetic group treated with curcumin 80 mg/kg were significantly higher than those in the control groups (*P*-value<0.001). Treatment with curcumin 130 mg/kg in the diabetic group caused a significant decrease in the NGAL and KIM-1 urine levels in comparison with the diabetic control group (*P*-value <0.001). The NGAL and KIM-1 concentration in plasma (Figures 3 C and D, respectively) was not significantly different among the studied groups (*P*-value >0.05). 

## Discussion

According to the results obtained in the current study, in addition to hyperglycemia, T1D causes weight loss and increases the serum concentration of urea, uric acid, and creatinine. In addition, our results showed that T1D induces oxidative stress in the kidney tissue and significantly increases the urinary excretion and renal expression of KIM-1 and NGAL. Meanwhile, in our study, curcumin treatment – in a dose-dependent manner – not only significantly improved the oxidative stress condition but also significantly reduced both urine levels and gene expression of KIM-1 and NGAL and corrected the urine parameters execration. 

In the present study, T1D significantly reduced body weight, but treatment with curcumin significantly improved it. Weight loss in rats with T1D has been documented by previous studies (28, 29). In insulin deficiency conditions, the cells cannot metabolize glucose; therefore, they use lipids and proteins as an alternative source of energy, and this leads to weight loss in diabetes. Consistent with our results, previous studies have shown that curcumin could modulate weight loss in diabetic rats (30, 31). Maintaining glucose and insulin levels, reducing the phosphorylation levels of the leptin receptor, and adiponectin induction are the main proposed mechanisms for improving body weight by curcumin (32). 

The present study revealed that STZ-induced diabetes increased the blood glucose levels, but treatment with curcumin significantly reduced the blood glucose levels of the diabetic group. Consistent with our results, Na *et. al.* demonstrated that curcumin increases muscle sensitivity to insulin signaling and thus stimulates glucose uptake by muscles and subsequently decreases blood glucose (33). In another study, it was found that curcumin stimulates the electrical activity of the pancreatic beta cells followed by membrane depolarization, insulin release, and finally glucose uptake (34). Another study carried out by Pari and Murugan showed that curcumin protects the pancreatic beta cells against the STZ-induced oxidative damage, and thereby preserves the level of insulin production (35).

Our results showed that T1D increases the levels of TG, LDL-C, and TC and reduces the HDL-C levels. However, treatment with curcumin 130 mg/kg improved this condition. A previous study revealed that STZ-induced diabetes caused a significant increase in TC and TG levels (36). The proposed mechanism in this regard is that insulin deficiency under diabetic conditions increases the levels of chylomicron and LDL-C, but decreases the activity of lipoprotein lipase, which finally results in hypertriglyceridemia (37, 38). Consistent with our results, another study demonstrated that curcumin treatment decreased the lipid profile in the diabetic group (39). It has been proposed that curcumin is involved in the peroxisome proliferator-activated receptor (PPARγ) activation and thereby results in the hypolipidemic condition (40). In addition, there is evidence that curcumin induces fatty acids synthesis through regulatory enzymes as well as specific transcription factors (41).

In the present study, T1D induced oxidative stress in the renal tissue, while treatment with curcumin improved oxidative damage in a dose-dependent manner. It was shown that hyperglycemia, caused by diabetes, increases oxidative stress through the overproduction of free radicals (42). Previous studies on diabetic rats have shown that curcumin improved oxidative stress (31, 39). The role of curcumin as an antioxidant and free radical scavenger, which has been confirmed in previous studies, can be due to the phenolic hydroxyl group or the methylene group of the β-diketone (heptadiene-dione) moiety (28). In addition, it has been reported that curcumin reduces the blood glucose levels and thereby increases the nicotinamide adenine dinucleotide phosphate NADPH/NADP ratio, which results in increased activity of glutathione reductase and subsequently elevates the GSH concentration (43). 

In the present study, type 1 diabetes caused a significant increase in the urinary levels and renal gene expression of KIM-1 and NGAL, as well as increased serum concentrations of urea, uric acid, and creatinine. However, treatment with curcumin – in a dose-dependent manner – significantly reduced KIM-1 and NGAL gene expression, urinary concentration of KIM-1 and NGAL, and corrected urea, uric acid, and creatinine excretion. KIM-1 is an extracellular protein and one of the markers of kidney damage that is up-regulated in type 1 diabetes with or without albuminuria (44). NGAL is a ubiquitous lipocalin iron-carrying protein which is bound to gelatinase in specific granules of the neutrophil involved in ischemic renal injury and repair processes as an early biomarker (45). In agreement with our results, in previous studies, it has been shown that under diabetic nephropathy, the renal expression of KIM-1 and NGAL is increased (46). In the current study, renal histological evaluation was not performed, but in the previous study, it was suggested that diabetic hyperglycemia results in damage to the extracellular matrix and hence increased vascular permeability, impaired blood flow, ischemia, hypoxia, and ultimately diabetic nephropathy (47). Accordingly, an increase in KIM-1 and NGAL gene expression as markers of renal ischemic injury was not unexpected in our study. On the other hand, treatment with curcumin decreased KIM-1 and NGAL gene expression levels. Furthermore, it has been shown that acute renal injury induced by glycerol increased KIM-1 gene expression, but treatment with curcumin improved it. It is suggested that antioxidant properties of curcumin are the main causes of the reduction of KIM-1 gene expression (48). Another study showed that gentamicin-induced renal injury caused a significant increase in the urinary excretion of KIM-1 and NGAL, while treatment with curcumin reduced the urinary excretion of KIM-1 and NGAL. Oxidative stress is proposed as the main cause of renal dysfunction, and curcumin improves these conditions due to its antioxidant properties (49).

Consistent with Alter *et. al.* (46), another interesting point in this study was that diabetes and treatment with curcumin affected only the urinary concentration (and not plasma concentration) of NGAL and KIM-1. Therefore, it can be concluded that urinary concentration of KIM-1 and NGAL may be used as a biomarker for the evaluation of diabetic nephropathy. 

Evaluation of the urinary factors in the present study also indicated that STZ-induced diabetes decreased the urinary excretion of urea, uric acid, and creatinine. However, treatment with curcumin significantly increased urinary excretion of these factors. Under diabetic conditions, inability to use carbohydrates as a source of energy and the use of alternative sources such as proteins significantly increases the blood concentrations of urea, uric acid, and creatinine (50). On the other hand, diabetic nephropathy decreases the kidneys’ ability to excrete these compounds. Treatment with curcumin improved diabetic conditions and improved diabetic nephropathy, thereby causing a significant increase in urinary excretion, uric acid, and creatinine, and decreased serum levels of these factors. One of the main important limitations of this study was that no histological evaluations were performed.

## Conclusion

Overall, based on the findings of the present study, curcumin was observed to reduce gene and protein expression of KIM-1 and NGAL and alleviate oxidative toxic stress in the kidney tissue of T1D rats. These data may have theoretical and practical implications concerning the management and treatment of nephropathy in diabetic patients.
